# Human VH4-34 antibodies derived from B1 cells are more frequently autoreactive than VH4-34 antibodies derived from memory cells

**DOI:** 10.3389/fimmu.2023.1259827

**Published:** 2023-12-15

**Authors:** Michelle E. Ray, Thomas L. Rothstein

**Affiliations:** Center for Immunobiology and Department of Investigative Medicine, Western Michigan University Homer Stryker M.D. School of Medicine, Kalamazoo, MI, United States

**Keywords:** B cells, antibody, autoreactive, CDR3, VH4-34, B1 cells, memory B cells

## Abstract

Human B1 cells produce natural antibodies characterized by overutilization of heavy chain variable region VH4-34 in comparison to other B cell populations. VH4-34-containing antibodies have been reported to be autoreactive and to be associated with lupus and other autoimmune dyscrasias. However, it has been unclear to what extent VH4-34 antibodies manifest autoreactivity in B1 cells or other B cell populations—in other words, are VH4-34 containing antibodies autoreactive wherever found, or mainly within the B1 cell population? To address this issue we sort purified single human B1 and memory B cells and then amplified, sequenced, cloned and expressed VH4-34-containing antibodies from 76 individual B cells. Each of these antibodies was tested for autoreactivity by HEp-2 IFA and autoantigen ELISA. Antibodies were scored as autoreactive if positive by either assay. We found VH4-34 antibodies rescued from B1 cells were much more frequently autoreactive (14/48) than VH4-34 antibodies rescued from memory B cells (2/28). Among B1 cell antibodies, 4 were HEp-2+, 6 were dsDNA+ and 4 were positive for both. Considering only HEp-2+ antibodies, again these were found more frequently among B1 cell VH4-34 antibodies (8/48) than memory B cell VH4-34 antibodies (1/28). We found autoreactivity was associated with greater CDR3 length, as expected; however, we found no association between autoreactivity and a previously described FR1 “hydrophobic patch”. Our results indicate that autoreactive VH4-34-containing antibodies tend to reside within the human B1 cell population.

## Introduction

B1 cells constitute a B cell subpopulation that has unique phenotypic, developmental, and functional characteristics in comparison to conventional B2 cells. B1 cells spontaneously secrete natural IgM antibodies without antigenic stimulation providing a baseline level of protection against foreign pathogens, unlike their conventional counterparts that adaptively react to antigen exposure (1–6). B1 cell natural antibodies include autoantibodies that can act as housekeepers to eliminate endogenous cellular debris and molecular toxins, ultimately providing homeostatic balance to the body (7).

The identity of human B1 cells was the subject of much controversy for a number of years. In mice, B1 cells are known to be derived from a separate progenitor than B2 cells, resulting in a separate lineage wherein CD5 expression distinguishes B1 and B2 cells (8, 9). Initially, this characteristic was carried over to human B1 cell identification (10). However, it was subsequently reported that the fraction of human B cells expressing CD5 is remarkably high (15-30% of circulating B cells) as compared to mouse, and that CD5 is present on several otherwise well-defined human B cell populations, including activated, transitional and pre-naïve B cells (11–15). As a result, CD5 is no longer considered to be a distinctive phenotypic marker for human B1 cells. To address the nature of human B1 cells, we utilized a reverse-engineering approach, in which we searched for human B cells that express key known functional characteristics of mouse B1 cells, chief among them spontaneous secretion of IgM, as well as strong stimulation of T cells and evidence of continual activation (16–20). This approach bore fruit with identification of CD27 and CD43 co-expression as important markers for human B cells that are functionally like mouse B1 cells (21, 22). The full phenotypic profile we defined consists of CD19+CD20+CD27+CD38low/modCD43+ among lymphocytes that are negative for T cell and activation markers (21–23). A recent report by Teichmann and colleagues confirmed this profile and showed that human B1 cells emerge early in development (24).

Beyond promoting homeostasis, autoreactive antibodies may reflect a critical break in immune system tolerance, and have been linked to several immune diseases and disorders, such as systemic lupus erythematosus (SLE) and rheumatoid arthritis (RA) (25). Autoreactive B cells are typically high producers of antibodies that have high affinities for self-epitopes (26, 27). In SLE and RA, autoantibodies present throughout the system react with DNA, RNA, histone, ribonucleoprotein, and other nuclear and non-nuclear antigens (28). Examination of autoreactive antibodies in SLE revealed that the heavy chain gene segment, VH4-34, is often an element in their composition (29–31). Many VH4-34-containing antibodies are directed against the I/i antigens of red blood cells, ssDNA, dsDNA, cardiolipin, bacterial LPS, chromatin, and rheumatoid factors (32–35). It is believed that VH4-34 is largely autoreactive without the presence of somatic hypermutation and often exists in a largely germline-like configuration (36). However, not all VH4-34-containing antibodies are autoreactive (37).

During study of the human B1 cell antibody repertoire we repeatedly found overutilization of VH4-34 in comparison to the antibodies produced by circulating memory B cells, pre-plasmablasts and plasmablasts (23, 38). Inasmuch as B1 cells often express autoreactive antibodies, we questioned whether B cells expressing VH4-34-containing antibodies that are autoreactive would all segregate to the B1 cell population, or whether a similar fraction of VH4-34 antibody-expressing B cells would be found to be autoreactive in every B cell population. To elucidate the answer to this question, we isolated peripheral blood mononuclear cells (PBMC) from healthy human donors, sorted individual B1 and memory B cells, single cell PCR amplified VH4-34+ antibodies, then cloned, expressed and purified these antibodies. The monoclonal antibodies produced were then tested against a series of antigens via ELISA and HEp-2 staining to determine the degree to which autoreactive vs non-autoreactive VH4-34-containing antibodies were found within the human B1 vs B2 cell populations. We found that B1 cells express more autoreactive VH4-34 antibodies than do conventional memory B cells, suggesting that autoreactivity is an intrinsic characteristic of the B1 cell repertoire.

## Materials and methods

### Donors and samples

Peripheral blood samples were collected at Western Michigan University Homer Stryker M.D. School of Medicine from healthy donors by venipuncture after informed consent was obtained, in accordance with a Western Michigan University Homer Stryker M.D. School of Medicine Institutional Review Board-approved protocol. De-identified samples were delivered to the lab where the blood was processed immediately.

### Blood sample processing

Peripheral blood mononuclear cells (PBMCs) were obtained through density gradient separation using Lymphocyte Separation Medium (Corning). The PBMCs were washed with standard wash medium (RPMI with penicillin/streptomycin and L-glutamine), and cell number and viability were determined with an automated cell counter (Digital Bio ADAM).

### B cell enrichment

B cells were enriched by magnetic bead selection using the EasySep™ Human CD19 Positive Selection Kit II (StemCell Technologies) according to the manufacturer’s instructions.

### Cell sorting

CD19+ cells were blocked with normal mouse serum (NMS) then stained with an antibody master mix to ensure consistent staining. The antibody staining cocktail was freshly prepared using optimized antibody concentrations consisting of anti-CD3-BV605 (Clone HIT3a; BD Biosciences 564712), anti-CD4-BV605 (Clone SK3; BD Biosciences 565998), anti-CD19-APC-Alexa700 (Clone J3-119; Beckman Coulter A78837), anti-CD20-Pacific Blue (Clone B9E9; Beckman Coulter A74777), anti-CD27-PE (Clone M- T271; BD Biosciences 557330), anti-CD38-PC5.5 (Clone LS198-4-3; Beckman Coulter A70205), anti-CD43-FITC (Clone DFT1; Beckman Coulter IM3264U), and Aqua LIVE/DEADTM viability stain. Stained cells were resuspended at a final concentration of 10 x 10^6^ cells/mL, and memory B cells and B1 cells were sorted on a BD Influx cell sorter.

### cDNA synthesis

Single B1 and memory B cells were sorted into individual wells of 96 well plates containing 20 µL/well of ice-cold lysis buffer (0.3125% IgePAL, 40 U/µL RNaseOut, 1 mg/mL carrier RNA, 0.0625 M DTT, and 5x First Strand/RT III Buffer). cDNA was synthesized in the original plate with a final volume of 24.25 µL using SuperScript III RT (200 U/µL), random hexamers (50 µM), dNTP mix (10 mM). Reverse transcription (RT) reaction occurred at 42°C for 10 mins, 25°C for 10 mins, 50°C for 10 mins, 94°C for 5 mins, after which products were immediately used or frozen at -20°C.

### Single cell RT-PCR and gene amplification

A master mix of 10x PCR buffer, 10 mM dNTP mix, HotStarTaq Plus, PCR grade water, 25 μM of chain-specific forward primer(s), and 25 μM of chain-specific reverse primer(s) was prepared on ice for the first round of semi-nested PCR. At the same time, the cDNA was concurrently thawed on ice if it was frozen. A volume of 23.75 μL of the master mix was added to each well of a new 96 well plate on ice, and 1.25 μL of cDNA was then added to the plate. Reverse transcription (RT) protocols and primers were adapted from Tiller et al. (39). IgH was amplified via two rounds of nested PCR and sequenced using the VH4-34 primer. To examine VH4-34-containing antibodies, a VH4-34 primer was used as a forward primer instead of the originally published 13-primer mix (39), after determining through a series of experiments that only using the one primer would not skew the number of VH4-34 amplifications that occurred, thereby streamlining the process.

Igκ amplification required an additional series of steps that differed from IgH and Igλ amplification to add proper restriction enzyme-specific cut sites during amplification. After the initial second round of semi-nested PCR with PanVK as the forward primer, the samples were sequenced using the PanVK primer and reamplified using newly aligned primers.

### Vector cloning

The amplified samples were purified using the Takara PCR/Gel purification kit and double digested with their respective restriction enzymes for 2 hours at 37°C with a 20-minute inactivation at 65°C. The heavy chain samples and AbVec2.0-IGHG1 vector were digested with Agel-HF and Sall-HF. Igκ and AbVec1.1-IGKC vector were digested with Agel-HF and BsiWi-HF. Igλ and AbVec1.1-IGLC2-Xhol vector were digested with Agel-HF and Xhol.

Digested samples and plasmids were purified via gel electrophoresis in 1% agarose gels. The samples were excised from the gels and purified using the Takara PCR/Gel purification kit. Purified digested samples and vectors were ligated using the Takara ligation system. Vector (20 ng) and insert (16 ng) were incubated together at 65°C for 2 minutes. The solution was cooled to 4°C on ice, and then the Takara solution was added. Afterward, the temperature was raised, and the samples were then ligated for 2 hours at 16°C.

OneShot competent *E. coli* cells were transformed with ligated samples via heat shock. 2 μL of ligation mix was added to 20 μL of *E. coli* cells and left on ice for 30 minutes. The samples were heat-shocked at 42°C for 30 seconds using a water bath and then immediately placed on ice again for at least 5 minutes. SOC medium (180 μL) was added to the samples, and they were horizontally shaken at 37°C in a Thermo Scientific MaxQ 8000 Shaker at 225 rpm for exactly 1 hour. Sample (200 μL) was spread on warm agar plates containing ampicillin to ensure only transformed cells grew, and the plates were placed, inverted, overnight in an incubator at 37°C.

Plasmid DNA was recovered from the bacterial pellet using the Qiagen MIDI prep kit. The OD A260/280 nm was measured to obtain the DNA concentration of the samples via the BioTek EPOCH 2 Plate Reader. The purified plasmid DNA was sequenced to ensure the correct orientation of the insert in the vector and to ensure the presence of VH4-34 in the heavy chain plasmids by using the SP6 universal primer from Genewiz.

### Transfection

Human embryonic kidney cell (HEK293T) cultures were prepared in advance for transfections. Cells were grown in complete DMEM (Dulbecco’s Modified Eagle Medium) 1x (with phenol red) with 4.5 g/L glucose, L-glutamine, and sodium pyruvate (Corning, 10-013-CV) supplemented with 10% heat-inactivated and filtered Fetal Bovine Serum (FBS) and 1x penicillin/streptomycin in 10 cm Falcon Tissue Culture-Treated Dishes.

Transfection medium consisted of phenol red-free and antibiotic-free DMEM supplemented with 4% FBS to efficiently purify the collected supernatant via the Bio-Rad Fast Protein Liquid Chromatography NGS system (FPLC). Cells were plated at 100,000 cells/cm^2^ (approximately 1.5 x 10^6^ cells/mL) to prevent the cells from becoming overconfluent the following day.

On the day of transfection, cells were observed under a microscope to ensure proper growth and health before transfection. Cells were co-transfected with IgH (heavy chain) and IgL (light chain) chain-specific plasmids using Lipofectamine 3000 following the manufacturer’s protocols for optimal transfection efficiency. Transfected plates were carefully placed in the incubator with 5% CO_2_ at 37°C. The first supernatant collection occurred two days post-transfection (day 3 of the procedure) and collection continued every two days until either the cells died, or 11 days post-transfection transpired. The supernatant was removed and filtered with a 0.22-micron filter. The transfection medium was carefully replaced, and the plates were placed back into the CO_2_ incubator between each collection. The collected supernatant from each day was pooled into one 50 mL falcon tube per sample and kept at 4°C until all collections were completed.

Additional transfections were carried out using ExpiHEK suspension cultures (ThermoFisher A14635) according to the manufacturer’s protocols and treated the same way for downstream purification.

### Concentrating the samples

The collected supernatant was placed in dialysis tubing with a 10kDa MWCO and covered with polyethylene glycol (PEG) 20,000. The samples were left in the PEG until approximately 10 mL of the sample was remaining and then removed from the tubing using a needle and syringe and stored in the syringe sealed with parafilm at 4°C until purified (no longer than two days).

### Sample purification

Samples were purified via FPLC and a Bio-Rad Bio-Scale Mini UNOsphere SUPrA Cartridge protein A column with 0.02 M sodium phosphate pH 7.5 as the wash buffer and 0.1 M glycine pH 2.5 as the elution buffer. Purified samples were collected in test tubes containing 100 μL of 1.0 M TRIS pH 8.8 with the Bio-Rad NGC Fraction Collector attached to the FPLC system. The fractions containing the purified antibodies were placed in a Thermo Scientific Slide-A-Lyzer Dialysis Cassette G2 (Prod# 87735) and put in a container filled with excess 1x PBS overnight at 4°C while being stirred to exchange the buffer the antibodies were stored in. The final samples were transferred to a Pierce Protein Concentrator (88517) and spun in a centrifuge until the sample volume was approximately 1 mL. The OD A280 nm was measured to obtain the concentration of the antibody via Epoch2.

### Determining reactivity and polyreactivity

ELISA kits from ORGENTEC Diagnostika GmbH (anti-dsDNA, ORG 604; anti-insulin ORG 520) and Abnova (anti-cardiolipin, KA0941; ANA Profile, KA1079) were used for basic qualitative binding of the cloned and purified monoclonal antibodies to determine autoreactivity to dsDNA, insulin, cardiolipin, and additional nuclear proteins. The manufacturer’s instructions were followed with additional adjustments in concentrations of sample added to the pre-coated plates made to optimize the procedure for purified antibodies compared to serum or plasma. Absorbances were collected via Epoch2, and data analysis was performed using GraphPad Prism versions 8 and 9 using the recommended 4-Parameter-Fit (4PL) with lin-log coordinates for the ORGENTEC anti-dsDNA and anti-insulin kits. Excel was used for the simple calculations for the Abnova anti-cardiolipin and ANA Profile kits.

Antibodies (10 µg) were tested for HEp-2 staining which was carried out according to the manufacturer’s instructions (Kallestad HEp-2 Slides, BioRad). The same dose was used for each monoclonal antibody to facilitate direct comparison.

### CDR3 analysis

CDR-H3 analysis was performed using IMGT/HighV-QUEST and NCBI IgGBlast to gather information from genetic sequences. The collected data was then analyzed using GraphPad Prism versions 8 and 9.

### Statistical analysis

Statistical analysis was performed using GraphPad Prism versions 8 and 9. Levels of statistical significance were determined via Fisher Exact tests and unpaired Mann-Whitney tests with significance taken as p ≤ 0.05.

## Results

### VH4-34 utilization

Overutilization of VH4-34 in antibodies expressed by healthy human adult B1 cells is well documented. Here B1 cells and memory B cells from 3 donors were sort purified following the gating strategy shown in **Supplementary Figure 1**. Antibodies expressed by these two populations were amplified and sequenced (**Supplementary Figure 2**). Of all (107) B1 cell antibodies, VH4-34 was expressed by 10.1%. Of all (65) memory B1 cell antibodies, VH4-34 was expressed by 3.56%. Thus, in this study, as in previous studies (23, 38), we found VH4-34 is overutilized in human B1 cell antibodies.

### Primer development

During this work, we adapted and altered the protocol that was originally outlined by Tiller et al. (39) to efficiently generate monoclonal antibodies. As we were only interested in one specific VH segment, there was no reason to use extra resources or complicate our work by continuing to use a 12 primer mix for VH segments when we were focused on amplification and expression of VH4-34-containing heavy chains. To validate the use of the VH4-34 primer alone, we ran several 96 well plates through the original protocol as well as our newly designed protocol and compared amplification success. In side by side comparison, samples from the new protocol amplified VH4-34-containing antibodies to the exact same extent (that is, in the same wells) as were amplified by the original protocol (data not shown). Using the VH4-34 specific primer method, 107 VH4-34-containing sequences were amplified from B1 cells (48 cloned), and 65 VH4-34-containing sequences were amplified from memory B cells (28 cloned).

### Antibody autoreactivity

**Figure 1:**
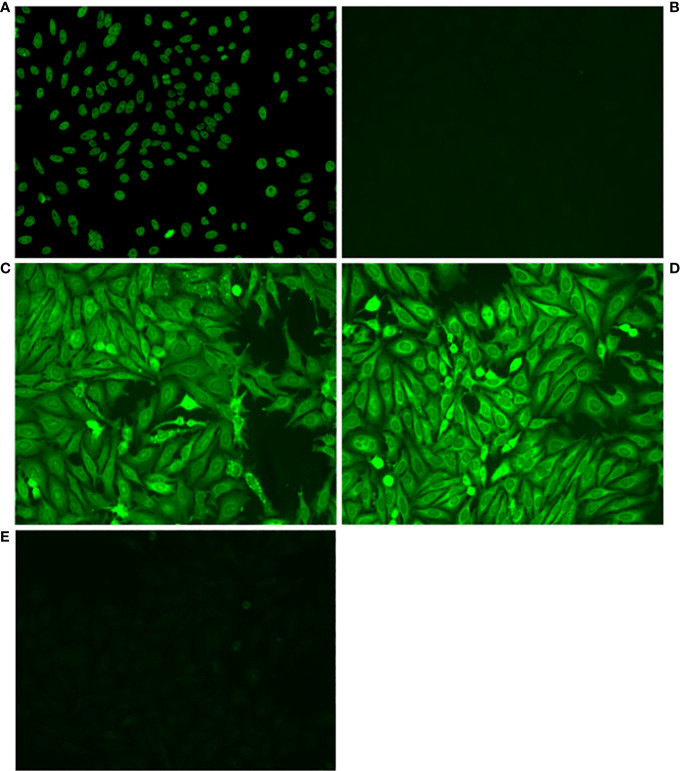
Some VH4-34 antibodies stain HEp-2 cells. Antibodies from single B1 and memory B cells, contributed by 3 healthy human donors, were amplified, cloned and expressed, and then tested for autoreactivity by indirect immunofluorescence assay on HEp-2 cells. Representative micrographs are shown of **(A)** Positive control, **(B)** Negative Control, **(C)** Antibody B1_3_C11_D2, **(D)** Antibody Mem_6_A10, **(E)** Antibody Mem_6_D7_IgG.

Amplified antibodies were cloned and expressed and then tested for autoreactivity by HEp-2 staining (**Figure 1**) and ELISA assays for recognition of a number of self-antigens. Positive results in any of these assays led to designation of an antibody as an autoantibody. Among all the cloned and expressed antibodies tested, we found autoantibodies were positive for HEp-2 staining (9) or anti-dsDNA ELISA (11), with 4 of these autoantibodies positive for both HEp-2 and anti-dsDNA, regardless of additional reactivities.

Among B1 cell-expressed antibodies that utilized VH4-34, we found 14 out of 48 (29%) were autoreactive, of which 8 were HEp-2+, 10 were dsDNA+, and 4 of these were positive in both assays (HEp-2+dsDNA+) (**Table 1**). Of the 3 B1 cell-expressed double positive HEp-2+dsDNA+ antibodies that were tested further, 2 were positive in 10 additional assays for autoantibodies (polyautoreactive), whereas of 8 B1 cell-expressed single positive (HEp-2+ or dsDNA+) autoantibodies that were tested further, none were positive on more than a single other ELISA assay. In direct contrast, among VH4-34 memory B cell-expressed VH4-34 antibodies, we found only 2 out of 28 (7.1%) were autoreactive, of which 1 was HEp-2+ and 1 was dsDNA+ (**Table 1**). The difference between the fraction of VH4-34 antibodies that were autoreactive in B1 cells vs memory B cells is statistically significant via Fisher’s exact test (p ≤ 0.05).

**Table 1:** VH4-34 antibodies that are autoreactive.

Sample Names	HEp-2	dsDNA	Insulin	Cardiolipin	Anti- RNP-70	Anti- RNP/Sm	Anti-Sm	Anti- SS-A	Anti- SS-B	Anti- Scl-70	Anti- Cemtromer B	Anti-Jo 1	Auto- reactive
B1_1_C6_lgM	+	–	–	–	–	–	–	–	–	–	–	–	yes
B1_2_D2_lgM	++	–	–	–	–	–	–	–	–	–	–	–	yes
Mem_6_A10_IgG	+	–	–	–	–	–	–	–	–	–	–	–	yes
Mem_6_C12_lgG	–	+	–	–	–	–	–	–	–	–	–	–	yes
B1_1_A12_lgG_D2	+	+++	+	+	+	+	+	+	+	+	+	+	yes
B1_2_A8_IgM_D2	–	+	–	–	–	–	–	–	–	–	–	–	yes
B1_3_A7_IgM_D2	+	–	–	–	–	–	–	–	–	–	–	–	yes
B1_3_C11_IgG_D2	+	+	–	–	–	–	–	–	–	–	–	–	yes
B1_1_H2_D2_IgG	+	–	–	–	–	–	–	–	–	–	–	–	yes
B1_1_F6_lgG	–	+++	ND	ND	ND	ND	ND	ND	ND	ND	ND	ND	yes
B1_2_A5_D2_IgG	–	+	ND	ND	ND	ND	ND	ND	ND	ND	ND	ND	yes
B1_1_B2_D2_IgG	–	+	ND	ND	ND	ND	ND	ND	ND	ND	ND	ND	yes
B1_2_B9_D2_IgM	–	+	ND	ND	ND	ND	ND	ND	ND	ND	ND	ND	yes
B1_3_C5_D2_IgM	++	++	ND	ND	ND	ND	ND	ND	ND	ND	ND	ND	yes
B1_1_C8_D2_IgG	+	+	+	ND	ND	ND	ND	ND	ND	ND	ND	ND	yes
B1_3_D3_D3_IgM	–	+	–	–	ND	ND	ND	ND	ND	ND	ND	ND	yes

"ND" not determined; "+" florescence barely above background; "++" mid level florescence observed; "+++" brightest florescence observed.

### Antibody sequence

**Figure 2:**
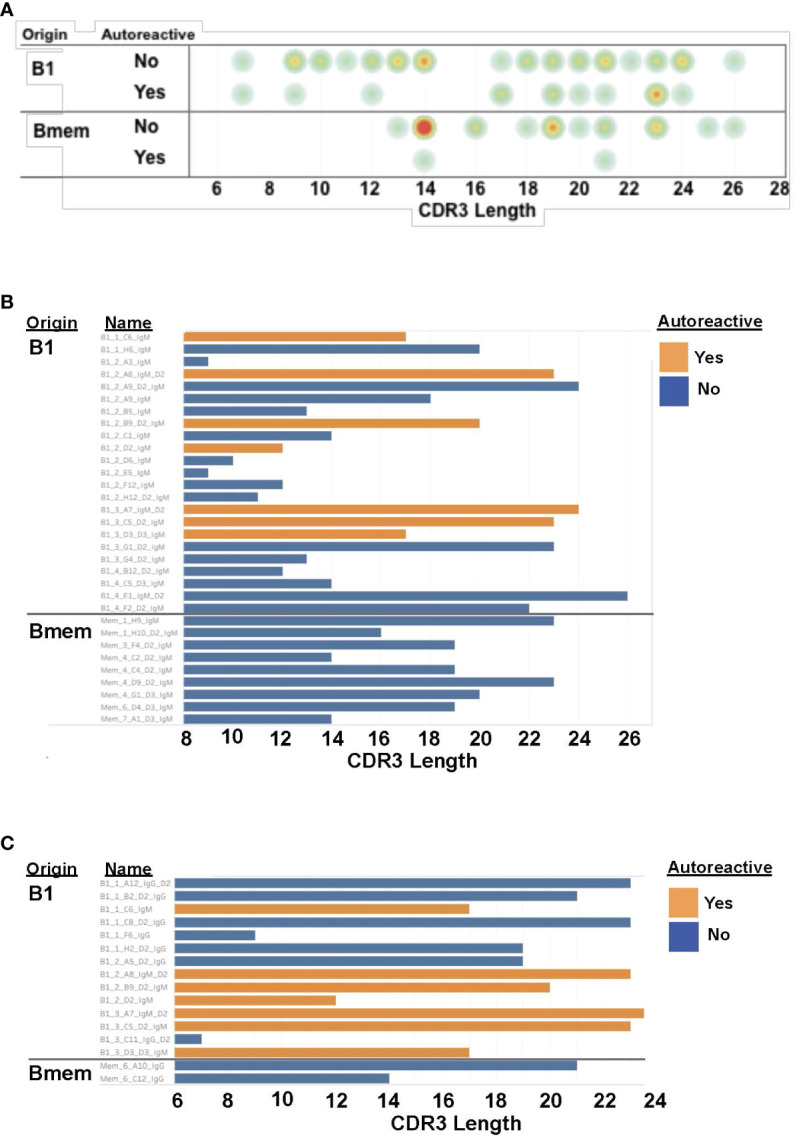
CDR3 regions of B1 cell autoantibodies encompass a range of lengths. **(A)** The CDR3 length for each antibody, categorized by cell of origin and autoreactivity, is shown as a circle with yellow/orange/red colors indicating more antibodies with that length. **(B)** The CDR3 length for each IgM antibody, categorized by cell of origin and autoreactivity, is shown as a bar graph. **(C)** The CDR3 length for each IgG antibody, categorized by cell of origin and autoreactivity, is shown as a bar graph.

Sequence information for autoreactive VH4-34 antibodies is shown in **Table 2**. We found utilization of DH and JH regions is heterogeneous among B1 and memory B cell VH4-34 autoreactive antibodies. Focusing on the antigen-binding CDR3 region, we found no statistical difference in CDR3 length between B1 cell-derived vs memory B cell-derived VH4-34-containing antibodies (**Figure 2**). However, VH4-34 autoreactive and non-autoreactive antibodies differed in CDR3 length in that CDR3 regions were longer in autoreactive antibodies (18.2 +/- 5.26 ranging between 7-24 amino acids) than in non-autoreactive antibodies (16.9 +/- 4.95 ranging between 7-24 amino acids), although this did not reach statistical significance.

**Table 2:** Characteristics of VH4-34 antibodies that are autoreactive.

Sample Names	Cell of Origin	Isotype of Origin	VH	DH	JH	FR1	CDR3 mut	Total Mut	CDR3	CDR3 length	Auto- reactive
B1_1_C6_lgM	B1	IgM	4-34	3-6	5-01	QVQLQQWGAGLLKPSETLSLTCAVY	0	0	ASNTDYVWGSYRPRGLL	17	yes
B1_2_D2_lgM	B1	IgM	4-34	3-22	4-02	QVQLQQWGAGLLKPSETLSLTCAVY	0	9	ARGPSGYYPFDS	12	yes
B1_1_A12_IgG_D2	B1	IgG	4-34	2-2	5-02	QVQLQQWGAGLLKPSETLSLTCAVY	0	0	ARALSGRYQLLYKRRAGYNWFDP	23	yes
B1_2_A8_IgM_D2	B1	IgM	4-34	2-2	6-02	QVQLQQWGAGLLKPSETLSLTCAVY	0	0	ARGTGCSSTSCHIRRYYYYGMDV	23	yes
B1_3_A7_IgM_D2	B1	IgM	4-34	2-15	6-02	LLKPSETLSLTCAVY	0	0	ARGVGYCSGGSCYSRYYYYYGMDV	24	yes
B1_3_C11_IgG_D2	B1	IgG	4-34	3-16	4-02, 5-02	QVQLQQWGAGLLKPSETLSLTCAVY	0	0	ARSLLRE	7	yes
B1_1_C8_D2_IgG	B1	IgG	4-34	2-2	5-02	QVQLQQWGAGLLKPSETLSLTCAVY	0	0	ARALSGRYQLLYKRRAGYNWFDP	23	yes
B1_3_D3_D3_IgM	B1	IgM	4-34	5-12	5-02	QVQLQQWGAGLLKPSETLSLTCAVY	0	10	ARGGSVSGNVVPTFLDR	17	yes
B1_1_H2_D2_IgG	B1	IgG	4-34	2-21	5-02	QVQLQQWGAGLLKPSETLSLTCAVY	0	0	ARGRGVTYCGGDCYPWFDP	19	yes
B1_1_F6_lgG	B1	IgG	4-34	5-24	4-02, 5-02	QVQLQQWGAGLLKPSETLSLTCAVY	0	1	ARGVMATSI	9	yes
B1_2_A5_D2_IgG	B1	IgG	4-34	3-22	4-02	QVQLQQWGAGLLKPSETLSLTCAVY	0	3	ARAPKALSGYPTRYTTLTA	19	yes
B1_1_B2_D2_IgG	B1	IgG	4-34	3-3	6-03	QVQLQQWGAGLLKPSETLSLTCAVY	0	0	ARGGVYDFWSGYNDYYYYMDV	21	yes
B1_2_B9_D2_IgM	B1	IgM	4-34	3-3	6-02	QVQLQQWGAGLLKPSETLSLTCAVY	1	9	ARGRGARTIFGVVRDYDLDV	20	yes
B1_3_C5_D2_IgM	B1	IgM	4-34	2-15	6-02	LLKPSETLSLTCAVY	0	0	ARGKVYCSGGSCYKVPYYYGMDV	23	yes
Mem_6_A10_IgG	Mem	IgG	4-34	3-3	6-03	QVQLQQWGAGLLKPSETLSLTCAVY	0	0	ARGGVYDFWSGYNDYYYYMDV	21	yes
Mem_6_C12_lgG	Mem	IgG	4-34	6-6	4-02	QVQLQQWGAGLLKPSETLSLTCAVY	0	0	ASPRQPGAARPYVR	14	yes

VH4-34 antibodies contained few mutations in CDR3 regions overall (**Table 2**). Among autoreactive antibodies, only 1/16 contained a single CDR3 mutation. Among non-autoreactive antibodies, only 8/60 contained any CDR3 mutation. Among autoreactive antibodies, 5/16 contained any VH mutations (numbering between 1 and 10), and among non-autoreactive antibodies, 47/60 contained any VH mutations (numbering between 1 and 32).

In VH4-34 antibodies, the presence of a germline “hydrophobic patch” in the VH4-34 antibody FR1 region (QWxxxxxxxxxxxxxxxAVY) has been reported to be associated with some forms of self-recognition (**Table 2**) (40). Among tested B1 cell antibodies, we found the FR1 “patch” in both autoreactive and non-autoreactive antibodies, being present in 12 of 14 B1 cell autoreactive antibodies (but not in all 14) and in 27 of 34 non-autoreactive antibodies. Thus, the presence of the “hydrophobic patch” sequence does not correlate with autoreactivity against dsDNA and HEp-2 cells in the structures examined here, as previously noted (29, 32).

## Discussion

Antibodies that incorporate the VH4-34 gene segment are strongly associated with autoreactivity and clinical autoimmunity (41). The finding that human B1 cells overutilize VH4-34 in comparison to other B cell populations would appear to be consistent with a homeostatic role for self-recognizing B1 cell natural antibodies. However, VH4-34 is incorporated in some antibodies expressed, albeit less frequently, by human B cells that are not B1 cells. This raises the question as to whether VH4-34-containing antibodies are equally autoreactive regardless of the B cell population they inhabit, or whether VH4-34 autoreactivity is a property of, and skewed toward, B1 cells. To address this issue, we sorted human B1 cells and memory B cells and then amplified, sequenced, cloned, expressed and tested 76 antibodies from single cells for autoreactivity. We found a statistically significant difference between B1 cells and memory B cells in which VH4-34-containing antibodies from the former were much more frequently autoreactive than VH4-34-containing antibodies from the latter. This strongly suggests that B cells with autoreactive B cell receptors tend to end up in the B1 cell pool, or that B cells with autoreactive B cell receptors tend to disappear from the memory B cell pool, or that other unknown elements play a role in preferentially skewing VH4-34-containing antibodies that are autoreactive toward the B1 cell population. Along these lines, it has been reported that certain light chains can suppress otherwise evident autoreactivity (42). We cannot say whether this mechanism is playing a role within the VH4-34+ memory B cell pool. Future experiments in which heavy and light chains are exchanged may provide insight into this issue. It is also notable that all amplified antibodies were expression cloned as IgG1, meaning antibodies from B cells expressing IgM, as well as from those expressing IgG, were constructed and tested as IgG1. It seems unlikely that IgG1 would strengthen antigen binding in comparison to IgM, but only comparison of the same binding domains cloned onto IgG1 vs IgM would address this issue conclusively (43). Despite these caveats, it is not unexpected that human B1 cells encode autoreactive antibody as this is well described in the murine system and there is preliminary evidence for this in our early work in the human system (21, 44–46).

We used two different methods here to determine whether cloned and expressed antibodies manifest autoreactivity: HEp-2 indirect immunofluorescence (IFA), and ELISA incorporating a variety of self-antigens, prominently including dsDNA. The HEp-2 immunofluorescent assay has been regarded as the gold standard for ANA testing in clinical settings (47) although the results can sometimes be difficult to standardize and interpret. In recent years, ELISAs, line immunoassays (LIAs), and bead assays have become more widely used in clinical and research settings with high sensitivity and specificity (48). It is with this in mind that ELISAs were used in addition to HEp-2 IFA to evaluate the autoreactivity of the cloned antibodies for these experiments. We labeled an antibody as autoreactive if it was positive in either IFA or ELISA. In that way we found a significant difference in the proportion of autoreactive antibodies among all tested antibodies for B1 cells vs memory B cells.

Antibody results from HEp-2 and ELISA screening did not fully align in our results. This inconsistency between tests has been noted before (49); however, despite imperfect overlap between the methods, both have been validated for determining presence of autoantibodies (50). The difference we documented for all autoreactive VH4-34 antibodies derived from B1 vs memory B cells is also true considering only antibodies that were HEp-2+, which amounted to 8 out of 48 B1 cell antibodies tested and 1 out of 28 memory B cell antibodies tested, as well as for those that were dsDNA+, which amounted to 10 out of 48 B1 cell antibodies tested and 1 out of 28 memory B cell antibodies tested.

Antibodies are composed of 6 CDRs, but it is the highly variable CDR-H3 region that is the central point of antibody-antigen interactions. It is the properties of the amino acids located in this region that dictate how each antibody responds to the myriad of antigens jeopardizing the body (51–53). It has been reported that longer CDR-H3 regions are associated with antibody autoreactivity (51, 54). The mean length, reported in literature, of the CDR-H3 is 15.2 ( ± 4.1) ranging between 1-35 amino acids (53). While there was no statistical difference in length of the CDR-H3 between B1 cell-derived vs memory B cell-derived VH4-34 containing antibodies, there was a trend toward a difference in CDR-H3 lengths between autoreactive and non-autoreactive antibodies. Although this difference is not statistically significant, our results are consistent with previous work associating CDR3 length with autoreactivity. However, our results did not support an association between the FR1 “hydrophobic patch” and antibody autoreactivity, as the consensus motif was widely distributed among VH4-34 antibodies examined here, both autoreactive and non-autoreactive, as shown before (29, 32).

This early work paves the way for additional analysis of VH4-34-containing antibodies derived from B1 cells compared to other subpopulations. A similar approach utilizing single cell sorting followed by amplification, sequencing, cloning, expression and testing of purified antibodies could further elucidate the extent to which autoreactive VH4-34 antibodies derived from B1 cells are responsible for autoreactivity in SLE and other VH4-34-associated human dyscrasias.

Notably, IgG is a recurring isotype among disease-associated autoantibodies. This has led to the presumption that clinical autoimmunity is associated with an adaptive B2 cell response. However, half of the B1 cell autoreactive VH4-34 antibodies were derived from B1 cells expressing IgG. This raises the possibility that clinically significant IgG autoreactive antibodies may arise from B1 cells in humans.

The need to further characterize autoreactive antibodies is evident in that specific B cell-targeted clinical therapies have become more and more common (35). Advanced characterization of the responsible autoantibody-producing subpopulation could guide more specific B cell-targeted therapies that do not eradicate all B cells (55–58).

## Data availability statement

The original contributions presented in the study are included in the article/**Supplementary Material**. Further inquiries can be directed to the corresponding author.

## Ethics statement

Peripheral blood samples were collected at Western Michigan University Homer Stryker M.D. School of Medicine from healthy donors by venipuncture, in accordance with a Western Michigan University Homer Stryker MD School of Medicine Institutional Review Board-approved protocol. De-identified samples were delivered to the lab where the blood was processed immediately. The studies were conducted in accordance with local legislation and institutional requirements. The participants provided their written informed consent to participate in the study.

## Author contributions

MR: Conceptualization, Data curation, Formal analysis, Investigation, Methodology, Visualization, Writing – original draft, Writing – review & editing. TR: Conceptualization, Data curation, Formal analysis, Funding acquisition, Project administration, Resources, Supervision, Writing – review & editing.

## Supplementary material

Supplementary Figure 1 Gating strategy designed and implemented to separate human B1 cells, memory B cells, and mature (naïve) B cells from whole blood. Pulse width and forward scatter allows for separation of singlets from doublets. Side scatter vs Aqua LIVE/DEAD dye is used to identify living cells. CD3/4/7 are used as a dump channel for T cells vs CD19^+^ B cells. CD19^+^CD20^+^ staining cells (B cells) are gated to exclude plasmablasts that are CD19^+^CD20^-^. CD19^+^CD20^+^CD27^hi^CD38^hi^ staining cells (preplasmablasts) are excluded. Among CD19^+^CD20^+^CD38^mod^ B cells, naïve, memory and B1 cell populations are single cell sorted based upon expression of CD27 and CD43, as indicated.

Supplementary Figure 2 Utilization of VH4-34. Antibodies were amplified from sort-purified B1 cells and memory B cells obtained from 3 different donors. Antibodies were cloned, expressed and then tested for autoreactivity.

## References

[1] Hayakawa K Hardy RR. Development and function of B-1 cells. Curr Opin Immunol (2000) 12:346–53. doi: 10.1016/S0952-7915(00)00098-4 10858035

[2] Hayakawa K Hardy RR Honda M Herzenberg LA Steinberg AD. Ly-1 B cells: functionally distinct lymphocytes that secrete IgM autoantibodies. Proc Natl Acad Sci U.S.A. (1984) 81:2494–8. doi: 10.1073/pnas.81.8.2494 PMC345088 6609363

[3] Rothstein TL Holodick NE. Activation of B-1 cells. Encyclopedia Immunol (2016) 3:237–43. doi: 10.1016/B978-0-12-374279-7.09021-4

[4] Baumgarth N. B-1 cell heterogeneity and the regulation of natural and antigen-induced igM production. Front Immunol (2016) 7:324. doi: 10.3389/fimmu.2016.00324 27667991 PMC5016532

[5] Hardy RR Hayakawa K. A developmental switch in B lymphopoiesis. Proc Natl Acad Sci U.S.A. (1991) 88:11550–4. doi: 10.1073/pnas.88.24.11550 PMC53173 1722338

[6] Herzenberg LA. Layered evolution in the immune system: a view from history. Ann N Y Acad Sci (2015) 1362:1–5. doi: 10.1111/nyas.12795 26096553 PMC4761344

[7] Rothstein TL. Natural antibodies as rheostats for susceptibility to chronic diseases in the aged. Front Immunol (2016) 7:127. doi: 10.3389/fimmu.2016.00127 27092140 PMC4823301

[8] Montecino-Rodriguez E Leathers H Dorshkind K. Identification of a B-1 B cell-specified progenitor. Nat Immunol (2006) 7:293–301. doi: 10.1038/ni1301 16429139

[9] Hayakawa K Hardy RR Herzenberg LA. Progenitors for Ly-1 B cells are distinct from progenitors for other B cells. J Exp Med (1985) 161:1554–68. doi: 10.1084/jem.161.6.1554 PMC2187623 3874257

[10] Hardy RR Hayakawa K. Development and physiology of Ly-1 B and its human homolog, Leu-1 B. Immunol Rev (1986) 93:53–79. doi: 10.1111/j.1600-065X.1986.tb01502.x 3096878

[11] Sims GP Ettinger R Shirota Y Yarboro CH Illei GG Lipsky PE. Identification and characterization of circulating human transitional B cells. Blood (2005) 105:4390–8. doi: 10.1182/blood-2004-11-4284 PMC1895038 15701725

[12] Lee J Kuchen S Fischer R Chang S Lipsky PE. Identification and characterization of a human CD5+ pre-naive B cell population. J Immunol (2009) 182:4116–26. doi: 10.4049/jimmunol.0803391 19299709

[13] Freedman AS Freeman G Whitman J Segil J Daley J Nadler LM. Studies of in *vitro* activated CD5+ B cells. Blood (1989) 73:202–8. doi: 10.1182/blood.V73.1.202.202 2462935

[14] Raman C Knight KL. CD5+ B cells predominate in peripheral tissues of rabbit. J Immunol (1992) 149:3858–64. doi: 10.4049/jimmunol.149.12.3858 1281192

[15] Wilson SM Wilkie BN. B-1 and B-2 B-cells in the pig cannot be differentiated by expression of CD5. Vet Immunol Immunopathol (2007) 115:10–6. doi: 10.1016/j.vetimm.2006.10.009 17098293

[16] Forster I Rajewsky K. Expansion and functional activity of Ly-1+ B cells upon transfer of peritoneal cells into allotype-congenic, newborn mice. Eur J Immunol (1987) 17:521–8. doi: 10.1002/eji.1830170414 2436924

[17] Sidman CL Shultz LD Hardy RR Hayakawa K Herzenberg LA. Production of immunoglobulin isotypes by Ly-1+ B cells in viable motheaten and normal mice. Science (1986) 232:1423–5. doi: 10.1126/science.3487115 3487115

[18] Zhong X Gao W Degauque N Bai C Lu Y Kenny J. Reciprocal generation of Th1/Th17 and T(reg) cells by B1 and B2 B cells. Eur J Immunol (2007) 37:2400–4. doi: 10.1002/eji.200737296 17683116

[19] Holodick NE Tumang JR Rothstein TL. Continual signaling is responsible for constitutive ERK phosphorylation in B-1a cells. Mol Immunol (2009) 46:329–30360. doi: 10.1016/j.molimm.2009.06.011 PMC2770333 19592097

[20] Karras JG Wang Z Huo L Howard RG Frank DA Rothstein TL. Signal transducer and activator of transcription-3 (STAT3) is constitutively activated in normal, self-renewing B-1 cells but only inducibly expressed in conventional B lymphocytes [see comments]. J Exp Med (1997) 185:1035–42. doi: 10.1084/jem.185.6.1035 PMC2196242 9091577

[21] Griffin DO Holodick NE Rothstein TL. Human B1 cells in umbilical cord and adult peripheral blood express the novel phenotype CD20+CD27+CD43+CD70. J Exp Med (2011) 208:67–80. doi: 10.1084/jem.20101499 21220451 PMC3023138

[22] Griffin DO Rothstein TL. Human B1 cell frequency: isolation and analysis of human B1 cells. Front Immunol (2012) 3:122. doi: 10.3389/fimmu.2012.00122 22654880 PMC3360193

[23] Quach TD Rodriguez-Zhurbenko N Hopkins TJ Guo X Hernandez AM Li W. Distinctions among circulating antibody-secreting cell populations, including B-1 cells, in human adult peripheral blood. J Immunol (2016) 196:1060–9. doi: 10.4049/jimmunol.1501843 26740107 PMC5351554

[24] Suo C Dann E Goh I Jardine L Kleshchevnikov V Park JE. Mapping the developing human immune system across organs. Science (2022) 376:eabo0510. doi: 10.1126/science.abo0510 35549310 PMC7612819

[25] Cashman KS Jenks SA Woodruff MC Tomar D Tipton CM Scharer CD. Understanding and measuring human B-cell tolerance and its breakdown in autoimmune disease. Immunol Rev (2019) 292:76–89. doi: 10.1111/imr.12820 31755562 PMC6935423

[26] Corsiero E Sutcliffe N Pitzalis C Bombardieri M. Accumulation of self-reactive naive and memory B cell reveals sequential defects in B cell tolerance checkpoints in Sjogren’s syndrome. PloS One (2014) 9:e114575. doi: 10.1371/journal.pone.0114575 25535746 PMC4275206

[27] Malkiel S Jeganathan V Wolfson S Manjarrez Orduno N Marasco E Aranow C. Checkpoints for autoreactive B cells in the peripheral blood of lupus patients assessed by flow cytometry. Arthritis Rheumatol (2016) 68:2210–20. doi: 10.1002/art.39710 PMC5523861 27059652

[28] Stollar BD. Autoantibodies and autoantigens: a conserved system that may shape a primary immunoglobulin gene pool. Mol Immunol (1991) 28:1399–412. doi: 10.1016/0161-5890(91)90042-I 1749388

[29] Richardson C Chida AS Adlowitz D Silver L Fox E Jenks SA. Molecular basis of 9G4 B cell autoreactivity in human systemic lupus erythematosus. J Immunol (2013) 191:4926–39. doi: 10.4049/jimmunol.1202263 PMC3816606 24108696

[30] Milner EC Anolik J Cappione A Sanz I. Human innate B cells: a link between host defense and autoimmunity? Springer Semin Immunopathol (2005) 26:433–52. doi: 10.1007/s00281-004-0188-9 PMC1431976 15633016

[31] Mockridge CI Chapman CJ Spellerberg MB Sheth B Fleming TP Isenberg DA. Sequence analysis of V(4-34)-encoded antibodies from single B cells of two patients with systemic lupus erythematosus (SLE). Clin Exp Immunol (1998) 114:129–36. doi: 10.1046/j.1365-2249.1998.00703.x PMC1905086 9764614

[32] Alcena DC Kobie JJ Kaminski DA Rosenberg AF Mattiacio JL Brewer M. 9G4+ antibodies isolated from HIV-infected patients neutralize HIV-1 and have distinct autoreactivity profiles. PloS One (2013) 8:e85098. doi: 10.1371/journal.pone.0085098 24386452 PMC3873436

[33] Bhat NM Lee LM van Vollenhoven RF Teng NN Bieber MM. VH4-34 encoded antibody in systemic lupus erythematosus: effect of isotype. J Rheumatol (2002) 29:2114–21. 12375320

[34] Cambridge G Moura RA Santos T Khawaja AA Polido-Pereira J Canhao H. Expression of the inherently autoreactive idiotope 9G4 on autoantibodies to citrullinated peptides and on rheumatoid factors in patients with early and established rheumatoid arthritis. PloS One (2014) 9:e107513. doi: 10.1371/journal.pone.0107513 25222933 PMC4164660

[35] Sanz I. Rationale for B cell targeting in SLE. Semin Immunopathol (2014) 36:365–75. doi: 10.1007/s00281-014-0430-z PMC4077421 24763533

[36] Pugh-Bernard AE Silverman GJ Cappione AJ Villano ME Ryan DH Insel RA. Regulation of inherently autoreactive VH4-34 B cells in the maintenance of human B cell tolerance. J Clin Invest (2001) 108:1061–70. doi: 10.1172/JCI200112462 PMC200949 11581307

[37] Reed JH Jackson J Christ D Goodnow CC. Clonal redemption of autoantibodies by somatic hypermutation away from self-reactivity during human immunization. J Exp Med (2016) 213:1255–65. doi: 10.1084/jem.20151978 PMC4925023 27298445

[38] Rodriguez-Zhurbenko N Quach TD Hopkins TJ Rothstein TL Hernandez AM. Human B-1 cells and B-1 cell antibodies change with advancing age. Front Immunol (2019) 10:483. doi: 10.3389/fimmu.2019.00483 30941130 PMC6433875

[39] Tiller T Meffre E Yurasov S Tsuiji M Nussenzweig MC Wardemann H. Efficient generation of monoclonal antibodies from single human B cells by single cell RT-PCR and expression vector cloning. J Immunol Methods (2008) 329:112–24. doi: 10.1016/j.jim.2007.09.017 PMC2243222 17996249

[40] Potter KN Li YC Capra JD. The cross-reactive idiotopes recognized by the monoclonal antibodies 9G4 and LC1 are located in framework region 1 of two non-overlapping subsets of human VH4 family encoded antibodies. Scand J Immunol (1994) 40:43–9. doi: 10.1111/j.1365-3083.1994.tb03431.x 8029642

[41] Tipton CM Fucile CF Darce J Chida A Ichikawa T Gregoretti I. Diversity, cellular origin and autoreactivity of antibody-secreting cell population expansions in acute systemic lupus erythematosus. Nat Immunol (2015) 16:755–65. doi: 10.1038/ni.3175 PMC4512288 26006014

[42] Wardemann H Hammersen J Nussenzweig MC. Human autoantibody silencing by immunoglobulin light chains. J Exp Med (2004) 200:191–9. doi: 10.1084/jem.20040818 PMC2212019 15263026

[43] Xia Y Janda A Eryilmaz E Casadevall A Putterman C. The constant region affects antigen binding of antibodies to DNA by altering secondary structure. Mol Immunol (2013) 56:28–37. doi: 10.1016/j.molimm.2013.04.004 23665381 PMC3687031

[44] Martin F Kearney JF. B-cell subsets and the mature preimmune repertoire. Marginal zone and B1 B cells as part of a “natural immune memory”. Immunol Rev (2000) 175:70–9. doi: 10.1111/j.1600-065X.2000.imr017515.x 10933592

[45] Palma J Tokarz-Deptula B Deptula J Deptula W. Natural antibodies - facts known and unknown. Cent Eur J Immunol (2018) 43:466–75. doi: 10.5114/ceji.2018.81354 PMC6384419 30799995

[46] Hardy RR. B-1 B cells: development, selection, natural autoantibody and leukemia. Curr Opin Immunol (2006) 18:547–55. doi: 10.1016/j.coi.2006.07.010 16879952

[47] Li Z Han R Yan Z Li L Feng Z. Antinuclear antibodies detection: A comparative study between automated recognition and conventional visual interpretation. J Clin Lab Anal (2019) 33:e22619. doi: 10.1002/jcla.22619 30030865 PMC6430365

[48] Mahler M Ngo JT Schulte-Pelkum J Luettich T Fritzler MJ. Limited reliability of the indirect immunofluorescence technique for the detection of anti-Rib-P antibodies. Arthritis Res Ther (2008) 10:R131. doi: 10.1186/ar2548 19000323 PMC2656233

[49] Copple SS Giles SR Jaskowski TD Gardiner AE Wilson AM Hill HR. Screening for IgG antinuclear autoantibodies by HEp-2 indirect fluorescent antibody assays and the need for standardization. Am J Clin Pathol (2012) 137:825–30. doi: 10.1309/AJCPICNFG7UCES1S 22523223

[50] Cressey R Pimpa S Chewaskulyong B Lertprasertsuke N Saeteng S Tayapiwatana C. Simplified approaches for the development of an ELISA to detect circulating autoantibodies to p53 in cancer patients. BMC Biotechnol (2008) 8:16. doi: 10.1186/1472-6750-8-16 18284706 PMC2275332

[51] Mroczek ES Ippolito GC Rogosch T Hoi KH Hwangpo TA Brand MG. Differences in the composition of the human antibody repertoire by B cell subsets in the blood. Front Immunol (2014) 5:96. doi: 10.3389/fimmu.2014.00096 24678310 PMC3958703

[52] Tsuji N Rothstein TL Holodick NE. Antigen receptor specificity and cell location influence the diversification and selection of the B-1a cell pool with age. J Immunol (2020) 205:741–59. doi: 10.4049/jimmunol.1901302 PMC7507122 32561570

[53] Zemlin M Klinger M Link J Zemlin C Bauer K Engler JA. Expressed murine and human CDR-H3 intervals of equal length exhibit distinct repertoires that differ in their amino acid composition and predicted range of structures. J Mol Biol (2003) 334:733–49. doi: 10.1016/j.jmb.2003.10.007 14636599

[54] Wardemann H Yurasov S Schaefer A Young JW Meffre E Nussenzweig MC. Predominant autoantibody production by early human B cell precursors. Science (2003) 301:1374–7. doi: 10.1126/science.1086907 12920303

[55] Sanz I Lee FE. B cells as therapeutic targets in SLE. Nat Rev Rheumatol (2010) 6:326–37. doi: 10.1038/nrrheum.2010.68 PMC3934759 20520647

[56] Huang W Quach TD Dascalu C Liu Z Leung T Byrne-Steele M. Belimumab promotes negative selection of activated autoreactive B cells in systemic lupus erythematosus patients. JCI Insight (2018) 3. doi: 10.1172/jci.insight.122525 PMC6171800 30185675

[57] Looney RJ Anolik J Sanz I. A perspective on B-cell-targeting therapy for SLE. Mod Rheumatol (2010) 20:1–10. doi: 10.3109/s10165-009-0213-x 19669389 PMC3927150

[58] Sanz I Anolik JH Looney RJ. B cell depletion therapy in autoimmune diseases. Front Biosci (2007) 12:2546–67. doi: 10.2741/2254 17127262

