# Supplementary figures and images for: Human VH4-34 antibodies derived from B1 cells are more frequently autoreactive than VH4-34 antibodies derived from memory cells
Source: Front Immunol. 2023 Dec 15;14:1259827. doi: 10.3389/fimmu.2023.1259827 (PMC10754998; doi:10.3389/fimmu.2023.1259827)

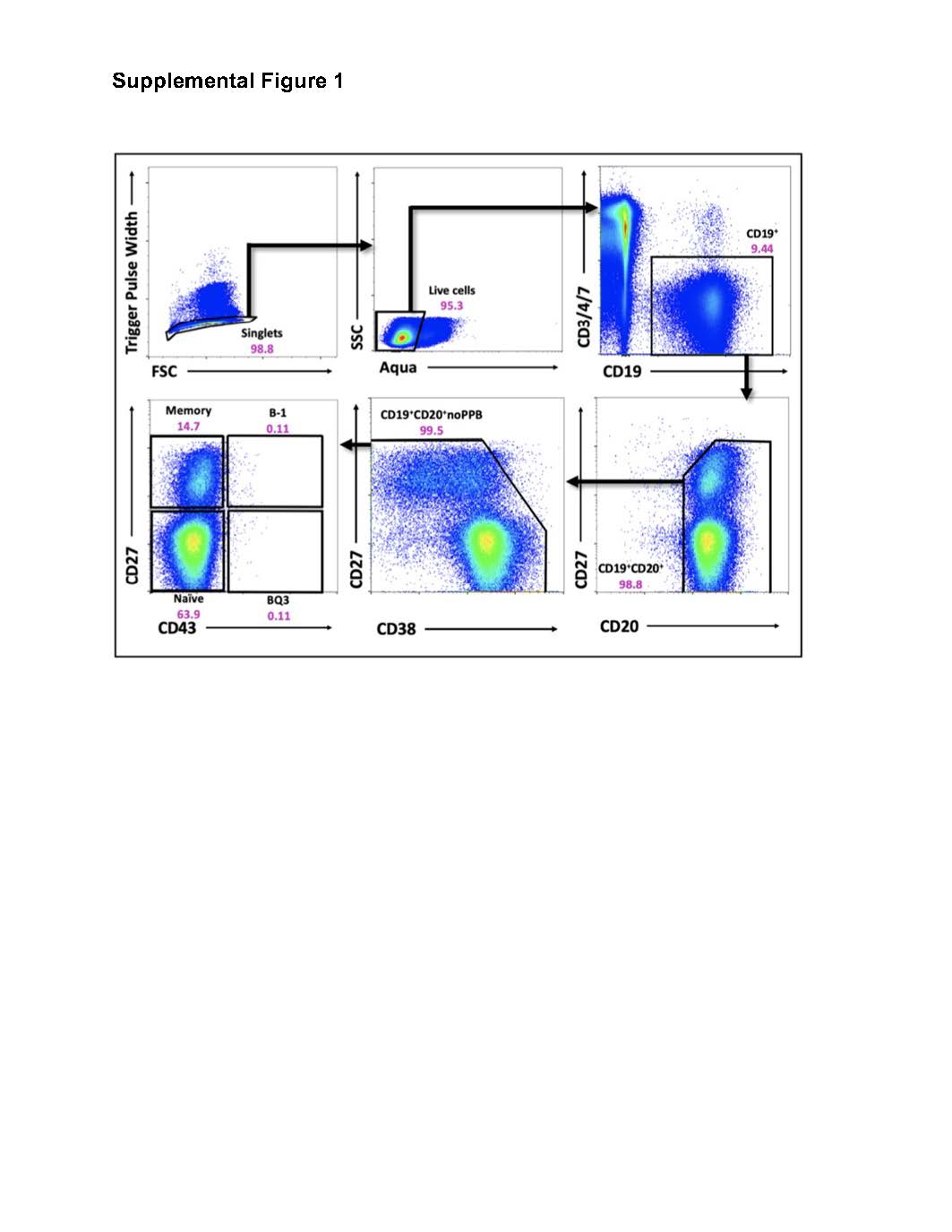

Supplement: Supplementary Figure 1 — Gating strategy designed and implemented to separate human B1 cells, memory B cells, and mature (naïve) B cells from whole blood. Pulse width and forward scatter allows for separation of singlets from doublets. Side scatter vs Aqua LIVE/DEAD dye is used to identify living cells. CD3/4/7 are used as a dump channel for T cells vs CD19+ B cells. CD19+CD20+ staining cells (B cells) are gated to exclude plasmablasts that are CD19+CD20-. CD19+CD20+CD27hiCD38hi staining cells (preplasmablasts) are excluded. Among CD19+CD20+CD38mod B cells, naïve, memory and B1 cell populations are single cell sorted based upon expression of CD27 and CD43, as indicated. [file Image_1.jpeg]

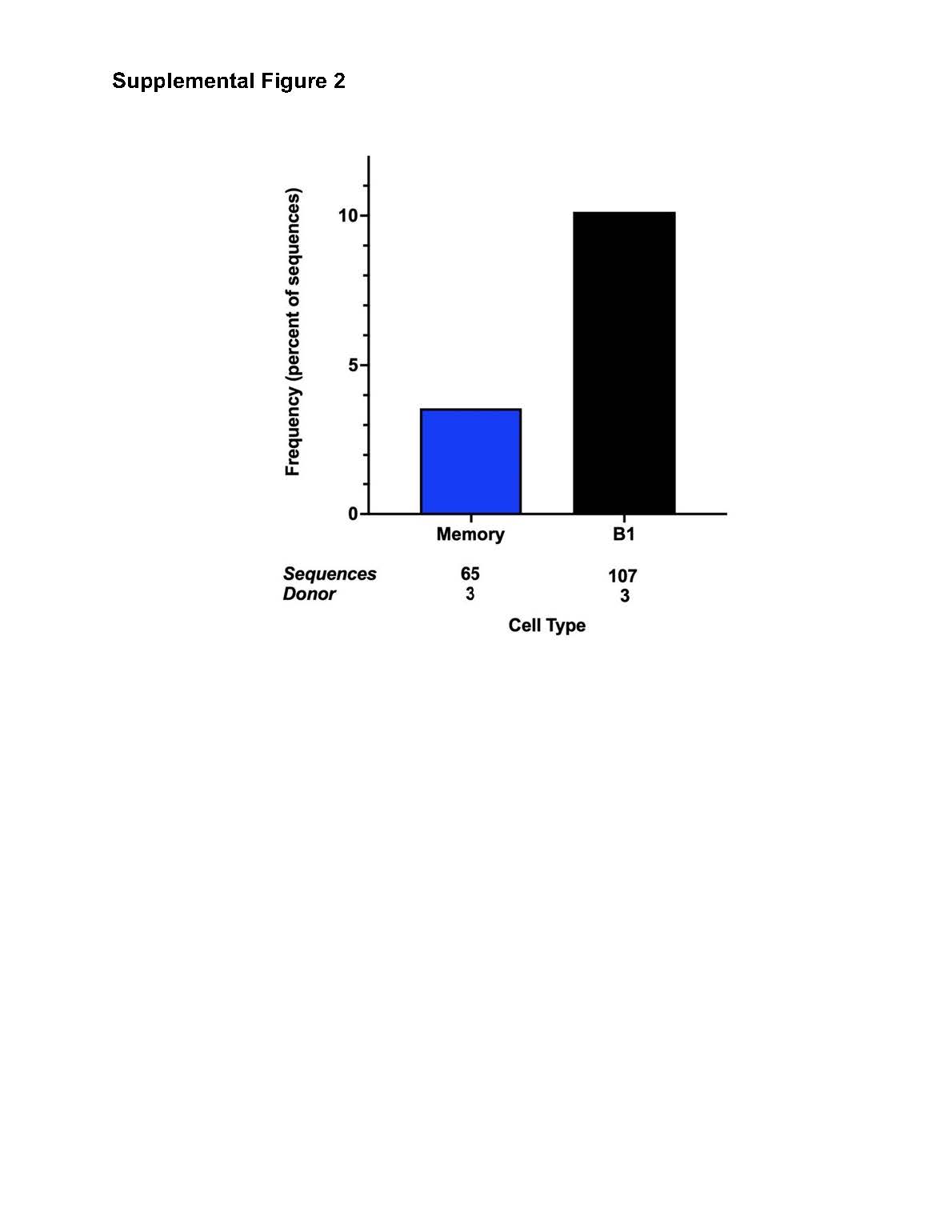

Supplement: Supplementary Figure 2 — Utilization of VH4-34. Antibodies were amplified from sort-purified B1 cells and memory B cells obtained from 3 different donors. Antibodies were cloned, expressed and then tested for autoreactivity. [file Image_2.jpeg]
